# A systematic review of the frequency, duration, type and effect of involuntary treatment for people with anorexia nervosa, and an analysis of patient characteristics

**DOI:** 10.1186/s40337-014-0029-8

**Published:** 2014-11-11

**Authors:** Loa Clausen, Allan Jones

**Affiliations:** Centre of Child- and Adolescent Psychiatry, Aarhus University Hospital, Skovagervej 2, DK-8240 Risskov, Denmark; Institute of Psychology, University of Southern Denmark, Campusvej 55, DK-5230 Odense M, Denmark

**Keywords:** Anorexia nervosa, Forced treatment, Involuntary treatment, Coercion, Compulsory treatment, Tube feeding, Treatment outcome

## Abstract

**Objective:**

Involuntary treatment of anorexia nervosa is controversial and costly. A better understanding of the conditions that determine involuntary treatment, as well as the effect of such treatment is needed in order to adequately assess the legitimacy of this model of care. The aim of the present study was to investigate the frequency and duration of involuntary treatment, the characteristics of this group of patients, the kind of involuntary actions that are applied and the effect of such actions.

**Review:**

Relevant databases were systematically searched for studies investigating the involuntary treatment of individuals diagnosed with anorexia nervosa.

**Results:**

The studies included in the review contained people treated in an inpatient setting for severe or severe and enduring anorexia nervosa. People that were treated involuntarily were characterised by a more severe psychiatric load. The levels of eating disorder pathology between involuntary and voluntary groups were similar and the outcome of involuntary treatment was comparable in terms of symptom reduction to that of voluntary treatment.

**Conclusion:**

Despite inconsistent findings, the comparable levels of eating disorder pathology observed between involuntary and voluntary patient-groups together with findings of higher co-morbidity, more preadmissions, longer duration of illness and more incidences of self-harm for involuntary patients suggest that involuntary treatment is not a reaction to the severity of eating disorder symptoms alone, but is most likely a response to the complexity of the patient’s situation as a whole.

## Introduction

Anorexia nervosa (AN) is a serious illness with the highest mortality of all psychiatric diseases [1]. Treatment usually consists of at least one year of therapy and even the most effective treatment-results still leave around 50% of patients unremitted after 5 years [2,3]. People with severe or severe and enduring anorexia nervosa often need inpatient treatment and some are treated involuntary, either under formal coercion, persuasion by relatives or professionals, or through parental consent (for children and some adolescents - due to differences in parental consent laws). Involuntary treatment in relation to patients with anorexia nervosa is usually characterised by involuntary admission or detention following voluntary admission. Involuntary treatment may in some cases involve procedures such as forced feeding, restraint or referral to a locked ward.

Several articles have been published discussing the ethical, clinical or legal aspects of involuntary treatment including case reports exploring the consequences of either using or not using involuntary treatment; some advocate for the use of involuntary treatment [4,5], while others take a more critical stance to the practice [6,7].

A study by Guarda et al. [8] reported that patients admitted to inpatient and day-care programmes perceived a high level of coercion even though the treatment was not categorized as involuntary. One third of the patients sampled (N = 139) had a perception of not endorsing treatment upon admission, however, after two weeks of nutritional rehabilitation nearly half (43%) of the “coerced” patients perceived the treatment as necessary. Two other studies surveyed attitudes of patients and mothers of patients diagnosed with anorexia nervosa and found that the majority of those interviewed deemed the use of involuntary treatment as appropriate when risk of mortality is high [9,10].

In order to better understand and adequately assess the utility and legitimacy of involuntary treatment of patients with anorexia nervosa, a systematic evaluation of the conditions that determine involuntary treatment and the effect of such treatment is needed. A systematic evaluation of the effect of involuntary treatment is however lacking. Previous reviews of involuntary treatment have not focused specifically on the frequency, type and effect of involuntary treatment [11-13]. The review by Russell [13] provides an overview of ethical and legal aspects of involuntary treatment along with a brief summary of three studies published at the time [14-16]. The review by Thiel and Paul [11] is written in German and includes three studies published in English and one study published in German [14-17], with a brief English version published by Thiels [12]. The three articles review part of the research in the area of involuntary treatment, however, a systematic and updated review of all studies evaluating the frequency, type, and effect of involuntary treatment as well as patient characteristics is needed.

### Aim of study

The aim of the present study is to systematically review the eating disorder literature in order to evaluate the frequency, duration, type and effect of involuntary treatment of people with anorexia nervosa as well as the characteristics of this group of patients.

## Review

PubMed, Embase, and Cinahl databases were searched using the following search strategy: (((”Coercion” OR”Treatment Refusal”) OR (Coercion[Mesh] OR Treatment Refusal[Mesh] OR”involuntary treatment” OR”forced treatment” OR”compulsory treatment” OR”forced feeding”))) AND (”Anorexia Nervosa”[Mesh] OR”Anorexia Nervosa”). No restrictions on date of publication were applied, with all studies published up until August 2013 included in the search. Articles published in the following languages were included: English, Danish, Swedish, and Norwegian. In addition, reference lists of relevant articles were searched for any articles that may have been missed by the initial search. Studies were included if they reported the effect (change in eating disorder symptoms e.g. BMI) of involuntary treatment of patients with anorexia nervosa. Single case studies were excluded.

## Results

**Figure 1:**
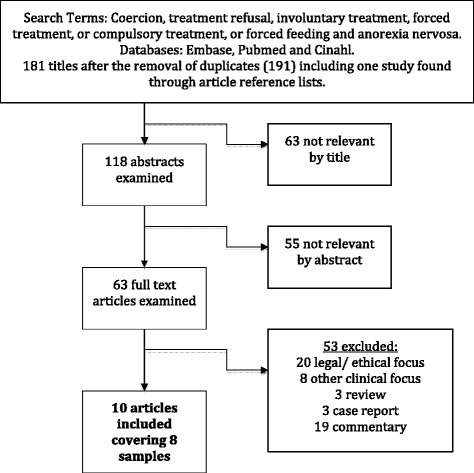
**A flowchart of the process of study selection.**

The initial search resulted in 191 articles. Figure 1 provides a flowchart of the process of study selection. Nine articles meeting the inclusion criteria were retrieved from the databases and an additional article was identified by searching the reference lists of relevant articles. Three out of the ten articles included for review contained the same sample, making a total of eight samples included in the review.

Table 1 provides a description of the eight studies including the following information: patient data (including type, age and number of patients), treatment type, treatment length, admission data, discharge data and follow-up data. Differences between involuntary and voluntary groups on all measures are shown in Table 2, and an assessment of the methodological quality of each study is presented in Table 3.

**Table 1:** **Description and results of studies included in the review**

**Study**	**Patients/Age/N**	**Treatment type**	**Treatment length**	**Admission data**	**Discharge data**	**Follow-up data**
**1. Ayton et al.** **[** 18 **]**	Adolescents	16 detained under section 3: 7/16 patients detained under VT, 9/16 before transfer to the clinic.	IT: 14 months	BMI: IT 16.6 ± 2.6, VT 14.2 ± 1.9	BMI: IT 19.6 ± 18.5 VT 18.5 ± 1.6	1 year follow-up
**UK**	Total N = 50	Nasogastric feeding:	VT: 8 months	Duration of illness: IT 3.8, VT 1.9	Menstrual period: 69% vs. 17%	N =41 (IT: 12/16 and VT: 29/34)
**Naturalistic 3 years inclusion**	IT: 16 (32%)	IT:69%, VT:12%		Age at onset: IT 12.5, VT 14.3	Overall ED outcome on M-R: IT 5.3 ± 3.1, VT 4 ± 2	Good outcome on weight and general functioning: IT 50%, VT 37.9%
	VT (parental consent): 34 (68%)			Purging type: IT 6%, VT 15%	HONOSCA: IT-28.1 ± 10.4, VT-18.3 ± 7.2	Readmissions:
				HONOSCA: IT 41.5 ± 4.8, VT 32 ± 5.0	CGAS: IT 47.2 ± 17.0, VT 36.6 ± 15.7	IT 16.7%, VT 34.5%
				Depression: IT 94%, VT 59% CGAS: IT 13 ± 6.5, VT 27 ± 9.0		Deaths: IT =0, VT =2
				Preadmissions: IT 88%, VT 29%		
				Low IQ: IT 19%, VT 3%		
				Abuse: IT 44%, VT 12%		
				Self-harm: IT 75%, VT 12%		
**2. Carney et al.** **[** 19 **-** 21 **]**	Adults and adolescents.	Guardianship or mental health committal. Some tube feeding (46% vs. 16%). Locked ward (42% vs. 1%) Otherwise not defined.	IT: 52 days	BMI: IT 13.2 ± 1.7, VT 14.03 ± 1.8	BMI: IT 14.9 ± 1.4, VT 15.4 ± 2.3	
**Australia**	Total N = 70		VT: 47 days	Purging type: IT 23%, VT 33%	Weight gain: IT 5.0 ± 6.6, VT 3.7 ± 5.3	
**Naturalistic 5 years inclusion**	70 patients with 96 admissions.		Mean: 49 days.	Preadmissions: IT 3.9 ± 3.4, VT 1.7 ± 2.3,		
	IT: 23 (33%)		40% <3 weeks.	Psychiatric comorbidity: IT 2 ± 1.6, VT 1 ± 0.9		
	VT: 47 (67%)					
**3. Griffiths et al.** **[** 14 **]** **, Australia**	Age 16–44 years	Guardianship.	IT: 15 weeks	BMI: IT 13.41 ± 1.76, VT 14.3 ± 2.2	BMI: IT 18.05 ± 2.14, VT 17.2 ± 2.9	3 of 15 IT patients reached at follow-up – 1 of 15 died, 4 not located.
**Naturalistic,**	Total N = 88	Otherwise not defined.	VT: 9 weeks	Binge/purge type: IT 60%, VT 43%	Weight gain: IT 10 kg, VT 8.7	
**IT cases from 4 units compared to VT**	IT: 15 (17%)			Residence - metropolitan: IT 67%, VT 50%	Reached target weight: IT 26.7%, VT 42%	
**from one of the units.**	VT: 73 (83%)			SES: IT 40%, VT 11%		
**4. Kondo et al.** **[** 22 **]**	Age 12–44 years	Involuntary admission for treatment and protection.	IT: 216 days	BMI: IT 15.3 ± 5.1,	Good outcome BMI:	
**Japan**	Total N = 70		VT: 70 days	VT 14.6 ± 8.0	IT 75%, VT 55%	
**Naturalistic**	IT: 8 (13%)					
	VT: 62					
**5. Laakman et al.** **[** 17 **]**	Age 16-39	Guardianship, forced feeding by tube until BMI = 17.5	Total 158 days	BMI:	BMI:	
**Germany**	Total N = 25		IT: 183 days	IT: 11.8	IT: 16.6	
**Naturalistic, all patients from 1 unit.**	IT: 11 (44%)		P: 166 days	P: 11.9	P: 16.7	
	Persuaded (P):		PC: 145 days	PC: 14.6	PC: 17.8	
	7 (28%)		VT: 98 days	VT: 12.1	VT: 18.8	
	Parental consent (PC): 2 (8%)					
	VT: 5 (20%)					
**6. Ramsay et al.** **[** 15 **]**	Adults	7/81 involuntary admitted, 30/81	IT: 113 days	BMI: IT 14.2 ± 2.7, VT 14.3 ± 2.4),	BMI: IT 18.7 ± 2.3,	5.7 years follow-up:
**UK **	Total N = 162	Detained after voluntary admission, 35/81 detained in other hospital before transfer to unit.	VT: 88 days	Bingeing history: IT 41%, VT 44%	VT 18.5 ± 2.0.	Deaths: IT 12.7% vs. VT 2.6%
**Time matched controls**	IT: 81 (50%)	No tube-feeding or physical restraint.		Vomiting history: IT 51%, VT 44%	Days to target weight (equal to treatment length:	9 out of 12 death certificates included AN
	VT: 81 (50%)			Laxative history: IT 49%, VT 49%	IT 113 days, VT 88 days	
				Preadmissions: IT 3.3, VT 1.8 Childhood abuse: IT 24%, VT 10%		
				Self-harm: IT 59% vs. VT 33%		
**7. Serfaty & McCluskey** **[** 23 **]**	Adults	Involuntary admitted only	Not specified	Mean BMI: 12.7	Mean BMI: 18.6	Mean Follow-up time 1 year
**UK**	Total N = 11	7/11 Nasogastric feeding.		Duration of illness 14.1 year	BMI >17.5 = 27%	Mean BMI: 17.9
**Naturalistic cases Patients in IT only**	All treated involuntary					BMI >17.5 = 45.5%
**8. Watson et al.** **[** 16 **]**	Adults	Involuntary admitted.	IT: 58 days	BMI: IT 17.4 ± 4.7, VT 18.4 ± 4.7 Preadmissions: IT 3, VT 1.4	BMI: IT 20.5 ± 3.8, VT 20.7 ± 3.6	
**USA**	Total N = 397		VT: 41 days	IQ: IT 91, VT 98	Weight gain: IT 18.8 ± 15.9, VT 13.9 ± 14.5 pounds	
**Naturalistic**	IT: 66 (17%)			Equal eating disorder symptoms, depression, and substance abuse.	Days to restored weight: IT: 58 days	
**7 years inclusion**	VT: 331 (83%)				VT: 41 days (Equal to treatment length)	

IT = Involuntary Treatment; VT = Voluntary Treatment; BMI = Body Mass Index; HONOSCA = Health of the Nation Outcome Scale for Children and Adolescents; CGAS = Children’s Global Assessment Scale: IQ = Intelligence quotient from the WAIS-R; SES = Socioeconomic Status; M-R = Morgan – Russell scale (eating disorder psychopathology).

**Table 2:** **Measures at admission, discharge and follow-up**

**Study** ^**(ref.)**^ **:**	**1** **[** 18 **]**	**2** **[** 19 **]**	**3** **[** 14 **]**	**4** **[** 22 **]**	**5** **[** 17 **]**	**6** **[** 15 **]**	**7** **[** 23 **]**	**8** **[** 16 **]**
**Admission/Discharge/Follow-up:**								
**BMI**	A↑*, D↑	A↓*, D↓,	A↓, D↑	A↑, D↑	A↑, D↓	A↓, D↑	A, D, F	A↓, D↓
**Duration of illness**	A↑*	A↑			A↑	A↑	A	A↑
**Age at onset**	↓*							
**Purging (bingeing) type**		A↓	A↑			A↑ (A↓)		
**Menstrual period**	D↑*							
**M-R**	A, D↑							
**HONOSCA**	A↑*, D↓*							
**BDI-2**	A↑*							
**C-GAS**	A↓*, D↑*							
**Preadmissions**	A↑*	A↑*	A↑		A↓	A↑*		A↑*
**Prior involuntary treatment**						A↑		
**Comorbid psychiatric disorders**	A↑*	A↑*				A↑		A↑
**IQ**	A↓							A↓*
**Physical or sexual abuse**	A↑					A↑*		
**Self-harm**	A↑*					A↑*		
**Substance abuse**								A↑
**SES**			A↓*					
**Treatment:**								
**Duration of treatment**	↑*	↑	↑*	↑*	↑	↑*		↑*
**Premature discharge**	↓							
**Frequency of re-feeding syndrome**		↑*						
**Frequency of tube feeding**	↑*	↑*			↑		?	
**Locked vs. open ward**		↑*						
**Treatment outcome:**								
**Weight increase**			D↑		D↑	D↑		D↑*
**Days to restored weight**						↑*		↑
**Reached target weight**			D↓					D↓
**Rate of weight restoration lb/week**								D↑
**Good outcome**	F↑*			D↑				
**Readmissions**	F↓							
**Death**	F↓					F↑*		

A = Admission; D = Discharge; F = Follow-up; ↑ = higher for the involuntary group; ↓ = lower for the involuntary group; * = significant differences between groups (p< 0.05); ? = not specified; BMI = Body Mass Index; M-R = Morgan – Russell scale (eating disorder psychopathology); HONOSCA = Health of the Nation Outcome Scale for Children and Adolescents; BDI-2 = Beck Depression Inventory −2; CGAS = Children’s Global Assessment Scale: IQ = Intelligence quotient from the WAIS-R; SES = Socioeconomic Status.

**Table 3:** Quality-assessment of study methodology*

**Study** ^**(ref.)**^ **:**	**1** **[** 18 **]**	**2** **[** 19 **]**	**3** **[** 14 **]**	**4** **[** 22 **]**	**5** **[** 17 **]**	**6** **[** 15 **]**	**7** **[** 23 **]**	**8** **[** 16 **]**	**Mean**	Max.**
**Reporting**	8	7	4	6	5	8	3	9	6.25	11
**External validity**	1	1	0	1	1	2	0	2	1.00	3
**Internal validity - bias**	2	3	1	1	1	2	1	2	1.63	7
**Internal validity – selection bias**	3	3	1	3	1	3	0	3	2.13	6
**Total score**	14	14	6	11	8	15	4	16	11.0	27

*Sub-scale scores on the *checklist for measuring study quality* by Downs & Black.^27^

Note: Higher scores indicate better methodological quality.

Reporting: degree to which information provided in the study is sufficient to allow an unbiased assessment of the findings (scale 0–11).

External validity: degree to which the findings from the study could be generalised to the population from which the study subjects were derived (scale 0–3).

Internal validity-bias: degree to which the study addressed biases in the measurement of the intervention and the outcome (scale 0–7).

Internal validity - selection bias: degree to which the study addressed bias in the selection of study subjects (scale 0–6).

**The highest possible mean score on the subscale.

The N of the eight samples ranged between 11 and 397 patients, with a combined total of 873 patients (231 treated involuntary and 642 voluntary). One study examined the outcome of involuntary patients only [23], while the remaining studies compared patients treated involuntary with patients treated voluntary.

Six studies reported the frequency of involuntary treatment in total inpatient samples, with frequencies ranging from 13-44%. When comparing treatment duration between involuntary and voluntary patients, five out of seven studies reported significantly longer treatment durations for involuntary patients [14-16,18,22]. The type of involuntary treatment applied was also examined, with forced tube feeding used in four of the studies [17-19,23]. Two of the studies evaluated the frequency of tube feeding, finding a significantly higher frequency among involuntary treated patients [18,19]. Only one study reported the non-use of forced tube feeding or of physical restraint [15]. Finally, the study by Carney et al. found use of locked wards and episodes of re-feeding syndrome to be significantly higher for involuntary patients [19].

### Involuntary and voluntary patient characteristics at treatment admission

All of the studies reported BMI at admission (see Table 2). However, BMI was not found to characterise group affiliation. Only the studies by Ayton et al. [18] and Carney et al. [19] found significant differences between involuntary and voluntary treated patient groups, with the two studies reporting diverging results (see Table 2). Involuntary treated patients displayed a more severe psychiatric load compared to voluntary treated patients. For example, four out of six studies found significantly more preadmissions among patients treated involuntary [15,16,18,19], two out of four studies reported significantly higher co-morbidity in involuntary patients at baseline [18,19], and duration of illness was found to be significantly higher for involuntary patients in the study by Ayton et al. [18] Involuntary patients measured significantly higher on episodes of self-harm in two of the studies [15,18], physical or sexual abuse in one study [15], and significantly lower on age at illness onset [18], CGAS [18], IQ [16], and socioeconomic status (SES) [14].

### Involuntary and voluntary patient characteristics at treatment discharge

All of the included studies reported patient data on weight at discharge, with overall findings indicating more similarities than differences between groups. No significant differences between groups on discharge BMI were observed in any of the studies (see Table 2), although the study by Watson et al. [16] found involuntary patients reported significantly greater increases in weight at discharge, and number of days needed to restore weight was found to be significantly longer for patients treated involuntary in the Ramsey et al. study [15].

### Involuntary and voluntary patient characteristics at follow-up

Only three out of the eight studies included follow-up data. The study by Serfaty & McCluskey reported that 45% of involuntary patients sustained their weight above BMI 17.5, however no data were available for voluntary patients in this study [23]. The study by Ayton et al. found that patients treated involuntary reported significantly better outcome at follow-up compared to voluntary patients, including better general functioning and normalisation of weight [18]. Finally, Ramsey et al. reported a significant difference in mortality between groups, with a 13% mortality rate for the involuntary group compared to 3% for the voluntary group [15].

## Discussion

### Frequency, duration and type of involuntary treatment

The frequency of involuntary treatment was reported in six of the studies reviewed and ranged from 13-44% of patients treated for anorexia nervosa. The differences between studies in the percentage of patients treated involuntary may partly be explained by differences between patient populations in eating disorder symptom-severity and co-morbidity. For example, in the 44% group [17] adults had a mean admission BMI of 11.8, while in a 17% group adults had a mean admission BMI of 17.4 [16]. However, when comparing mean BMI at admission with the percentage of involuntary treatment across all studies the picture is less clear. Due to the controversial nature of forced intervention, cultural, organisational and procedural/legal differences between institutions and countries must also be considered, as decisions regarding treatment may be based more on tradition and local regulations and procedures and less on symptom severity or evidence of treatment efficacy [24,25]. A study examining compulsory admission regulations and procedures across countries in the European Union reported that the differences in regulations and procedures across countries were the most significant source of variance [26]. Also, in addition to patient characteristics, hospital characteristics were shown to be an independent predictor of involuntary treatment in a Swiss population [27].

Information on the type and process of involuntary treatment was very limited in the studies included for review. Type of involuntary treatment can include, but is not limited to, use of physical restraint (including use of straps), locked wards, forced feeding and forced medication. Four of the studies reviewed reported the use of tube feeding [17-19,23], and one study explicitly reported the non-use of tube feeding and physical restraint [15]. Overall, studies show that there is a higher frequency of tube feeding for involuntary patients, however the notable lack of information on type of involuntary treatment needs to be addressed in future studies.

### Effect of involuntary treatment

Treatment under involuntary conditions seems to last longer and include more tube feeding, more re-feeding syndrome and use of locked wards. Interestingly, the overall effect of involuntary treatment appears to be comparable to voluntary treatment. When comparing group differences in BMI at discharge across studies the results are inconclusive with any variance in treatment outcome probably masked by treatment continuing until target-weight was reached [14-18]. Weight increase was shown to be significantly higher [16] and time taken to restore weight significantly longer for involuntary patients [15]. The follow-up data on treatment effect is sparse and results are mixed making any conclusions on the long-term effect of involuntary versus voluntary treatment difficult. The higher mortality rate observed in the Ramsey et al. [15] study compared to the Ayton et al. [18] study for involuntary patients is likely due to the difference in mean age across samples. Studies evaluating the long-term effect of voluntary versus involuntary treatment should control for age as outcome measures may differ between adolescent and older-adult populations.

The effect of different types of involuntary treatment is also unclear. To the best of our knowledge there is only one randomised controlled trial in which the efficacy of an “involuntary-type” treatment modality was examined (the study was not included in the review as the treatment was not reported as involuntary). Rigaud et al. [28] randomly assigned inpatients to either combined tube-feeding and normal meals or normal meals only. The combined tube feeding and normal meals group were found to gain more weight, display fewer bingeing episodes and have longer relapse-free periods compared to the normal meals only group [28].

### Determinants of involuntary treatment/patient characteristics

When looking at severity in eating disorder symptoms at admission across studies the results are mixed with more similarities than differences observed between involuntary and voluntary groups. Duration of illness was a significant predictor of involuntary treatment for adolescent patients in the Ayton et al. [18] study, with four other studies finding duration of illness to be higher for involuntary patients – although not reaching significance [15-17,19]. Longer duration of illness, together with findings of higher psychiatric co-morbidity, more preadmissions, and more incidences of self-harm for involuntary patient groups, suggest that involuntary treatment is not a reaction to the severity of eating disorder symptoms alone, but is most likely a response to the complexity of the patient’s situation as a whole. BMI at admission was expected to partly determine treatment choice, however, this was not clearly shown in the present review. Involuntary treatment is initiated on the basis of either the dangerousness criterion or the need-for-treatment criterion indicating a more severe status. If, as the current review suggests, involuntary treated patients are not predominantly more somatically threatened by eating disorder pathology than voluntary patients, then what other factors are being taken into consideration when prescribing this forced intervention? Somatic problems such as cardiac arrhythmia or severe electrolyte imbalance, or threat due to suicidal thoughts/ideation could all be reasons for involuntary treatment, however, none of these problems are reported in any of the studies reviewed.

### Study limitations and concluding remarks

Due to the relatively small number of studies evaluating involuntary treatment in anorexia nervosa it is difficult to know if the results reported in the present review are representative of involuntary treatment in general. The majority of studies included compulsory admission and involuntary treatment cohorts. Information on treatment admission was limited in two of the studies [14,23] making it unclear as to whether treatment admission was involuntary for all patients or whether some patients were admitted on a voluntary basis and then treated involuntary. In two of the studies [14,18] it was noted that is was possible for voluntary treatment to precede involuntary treatment, but further information was not made available. Also, the studies reviewed provide sparse information on the involuntary treatment applied. In general the methodological quality of the studies was low. This was due in part to the reliance on retrospective data collection (from medical journals) and naturalistic research designs. Table 3 shows an assessment of the methodological quality of each study (measured using the *checklist for measuring study quality* by Downs and Black [29]) with overall scores indicating issues with external validity, internal validity, and selection bias. Moreover, with the exception of the studies by Ramsey et al. [15] and Watson et al. [16], studies included in the review lacked sufficient statistical power to detect medium (clinically relevant) effect sizes due to limited sample sizes in the involuntary treatment groups. The overall low level of methodological quality in the studies included in the review is a limitation and must be taken into consideration when interpreting the present findings.

The present findings reflect the complexity of the situation that people with severe anorexia nervosa find themselves in. There is a clear need for large, well designed studies reporting the frequency, type and effect of involuntary treatment in relation to voluntary treatment, as well as the characteristics of patients admitted to involuntary treatment. A better understanding of the determining conditions and effect of involuntary treatment may aid in developing clearer guidelines in the use of this forced intervention.

While this article was under review a comparable article was published [30]. The comparable article includes a more recent search, and uses different inclusion criteria than the present study with less focus on predictors and the effect of involuntary treatment, and more focus on clinical analysis. Despite these differences, the overall results and conclusions are similar across studies.
